# Dynamic Assessment of Binocular Eye Movement Coordination: Norms and Functional Implications

**Published:** 2014

**Authors:** Erik Viirre

**Affiliations:** Department of Neurosciences, School of Medicine, University of California, San Diego, CA 92037, USA

**Keywords:** Binocular Eye Movement Coordination, Functional Implications, Eye Alignment, Intranuclear Ophthalmoplegia

## Abstract

Alignment of the two eyes is controlled by a finely tuned, fast acting system with components within the brain. Assessment of binocular alignment has classically been done statically. Eye positions are assessed in primary position and at eccentric angles to interpret the functional status of the oculomotor nerves and muscles. However, assessment of dynamic eye alignment, the coordination of the eyes during eye movements, has been less commonly carried out and has not been formalized with population norms. Clinicians are aware of slow eye movement dynamic alignment changes, such as that clinically observed in Intranuclear Ophthalmoplegia. But assessment of eye alignment during rapid eye movements, such as saccade or pursuit has not been part of neuro-ophthalmologic assessment. With the advent of inexpensive, high resolution recording systems, both eyes can be simultaneously recorded and their coordination during movement compared. Thus, we now have an opportunity to provide a laboratory based objective measurement of a gamut of binocular coordination systems. Recent research in humans has demonstrated increased variability of binocular coordination during divided attention. Variability is an interesting statistic that can be sensitively assessed in the velocity domain without extensive gaze position recalibration procedures during recording over long intervals. Variability can thus be used as a robust, long-term eye movement parameter with minimal intrusiveness to the subject. It is proposed that population studies of binocular coordination during eye movements be carried out to determine neurologic norms so that conditions such as brain injury and others can be assessed with a functional tool with objective parameters.

## INTRODUCTION

Alignment of the eyes is maintained by the brain with a sophisticated system that analyses visual information and then finely controls the fast acting extra-ocular muscles. The system is remarkably precise and it keeps working as we grow, age and encounter disease. Subtle changes in the eye movement coordination dynamics may result in symptoms such as eye-strain, and with institutionof clinical population norms, measurement of those dynamics may also become reliable objective indicators of neural dysfunction.

 Misalignment of the two eyes as a movement control disorder of the brain is most commonly evident in those people with strabismus. An eye that loses or does not develop vision often “wanders” and can be readily observed to not align with the seeing eye. The misalignment can be monitored as the person attempts to fixate on an object, such as an observer’s facial features. An extra-ocular muscle problem can also cause a misalignment that is also easily seen by a casual observer. Current clinical examination methods of people with strabismus generally include review of the visual acuity of both eyes and then stationary measurements of the magnitude of the misalignment in primary position as well as in positions of eccentric gaze. The combination of assessment of acuity and alignment in various gaze directions can help the determination of whether a sensory deficit or a movement control defect may be the cause of the misalignment. 

 However, primary visual input and eye muscle motor output are not the only brain activities active in alignment of the eyes. There is a highly complex integrated system working throughout the brain that does so. The system extends from image capture in the retina all the way through the vision system, passing through the higher order image analysis functions of the brain to the target selection and fixation maintenance systems out to the oculomotor control centers in the brainstem and finally to the muscle control motor neurons. There are ongoing calibration and stability functions acting on the eyes with reflex systems such as the Vestibulo-Ocular Reflex (VOR). Examination of the eyes while they move is an important neurological assessment tool. Clinically we know about participation of the whole brain in eye movement control based on observations of the moving eyes in various conditions of neurologic disease such as: saccadic pursuit observed in cerebellar disorders or altered main-sequence velocity of saccades in drug intoxication. We also have the example of visibly slowed ocular alignment in inter-nuclear Ophthalmoplegia (INO) in Multiple Sclerosis (MS) of the brain stem. Abnormalities such as nystagmus and slowed eye movements recorded with electronics give us sensitivity for detection of disease throughout the brain beyond the static alignment of eyes observed in strabismus. In the previous era of expensive electronics, eye movement recording systems often included recordings from one eye only. Now, as technology has improved to the level of eye recording systems on smart phones, the incremental expense of recording both eyes rather than just one is trivial. New levels of capability of technology of eye movement recording now afford us the opportunity to assess quantitatively more of the eye movement control apparatus in the brain through assessment of binocular coordination dynamically. By measuring both eyes during movement and comparing their dynamics, we will have a new sensitive measurement tool to detect neurologic dysfunction and even subtle changes in neurologic state such as those that occur with changes in attention. 

 In an example of binocular dynamic assessment, we examined the variability of alignment angle during a driving simulation task (1,2). The convergence angle should have remained constant, as the task involved viewing objects on a flat plane (a computer monitor). During the single required task of “driving” the simulation, the vergence angle remained relatively constant over intervals of minutes, even with the intrusion of hundreds of saccades. However, when an auditory task was introduced during the simulation (listening for numbers, performing mental addition on them and speaking a result) the variability of the vergence angle increased dramatically, despite no change in the required visual task. With increased difficulty of the auditory task, the vergence variability increased. Ironically, the simulation task, which took place on a 2D environment, readily enabled us to demonstrate a 3D coordination change in the oculomotor system. Similarly, Bucci (3) and Gaertner (4) demonstrated dynamic oculomotor changes during reading in children with dyslexia and with vergence difficulties. Importantly, like the driving simulation task, reading takes place on a plane a constant distance from the eyes, thus deviation from the constant convergence angle is easily measured. Curiously, an important question is whether prolonged viewing of flat planes such as books or displays is a “natural” condition.

 Because of the high precision of the oculomotor system and the low variability of its performance among humans, well characterized performance envelopes of eye movement parameters have been established. Perhaps the most well-known is the “main sequence” of saccade peak velocity relative to saccade size, first described by Bahill (5). We can predict very well what a given saccade’s peak velocity should be based on the angular distance between start and finish positions. The main sequence curve is programmed into eye movement diagnostic systems and can be used to detect off-nominal performance of the eyes in motion. For example, diazepam will reduce the peak velocity of a given saccade size (6). It is proposed that analogous values to the main sequence be determined for binocular coordination in populations of people with normal binocular vision. These values would be of binocular coordination during intervals with frequent eye movements. Indeed these coordination metrics should be captured during existing routine oculomotor assessments: horizontal and vertical saccade arrays, pursuit target tracking, optokinetic stimuli, VOR testing and even positional eye movement assessments and caloric testing. The hypothesis is that during all of these tasks the convergence angle should remain constant as the tasks are done with targets on a plane a constant distance from the eyes. Thus any variation, as we detected in the driving task and Bucci (3) and Gaertner (4) determined in the reading task should be immediately evident. With bi-ocular calibration of each eye’s movements statistics analogous to the “main sequence” will be established for normal populations under constant conditions. Once norms have been well established, then study of conditions where coordination might be disrupted will be rapidly executed and ultimately oculomotor laboratory systems will contain established clinical norms with which individual patients can be assessed. We will have progressed from the careful static clinical assessment of strabismus surgeons and MS neurology experts to an objective laboratory program.

## HYPOTHESES

For this project of review of binocular coordination norms, the hypotheses are:

Binocular coordination norms can be:

-Readily established

-Simple, easy to execute (maybe on top of existing approved oculomotor tests)

- Sensitive to common neurological states: fatigue, drugs, etc.

- New objective functional metrics of neurological disorders such as post-concussion

- A real-time or near real-time means of assessing orthoptic exercises

- A new tool for assessing effects of activities of daily living, such as computer use.

## DISCUSSION

To establish the population norms of convergence angle variability, a variety of conditions need to be met. Obviously a bi-ocular eye movement recording system with independent calibration of each eye will be needed. For the test stimuli, using the standards for existing FDA approved oculomotor batteries is the most sensible. Targets at a prescribed distance (typically one meter) on calibrated screens will be used. Then the batteries of tests: saccades, pursuit, OKN etc. will be carried out. Since there will be no cost other than a small increase in computation effort, recording of both eyes during VOR testing, positional testing, Dix-Hallpike procedures and positional testing with eyes open and eyes closed will be carried out as well. Movements horizontally, vertically and obliquely will be assessed.

**Binocular Coordination Statistic: Vergence Variability**

The appropriate statistic will be Vergence Variability. Working on a flat plane, the average convergence angle change from interval to interval should be zero. Thus the amplitude of the deviation from zero (convergence or divergence) will be the appropriate metric of variation. The variability can then readily be compared from condition to condition. Importantly, even if there are occasional baseline shifts in measured convergence angle, the variability statistic from interval to interval will be a robust enough metric of oculomotor control system status. 

 Beyond the measurement of both eyes and the convergence variability during movements on a flat plane in front of the subject, one additional task might be contemplated: measurement of active convergence and divergence. However, such a measurement would involve additional complexity, such as a 3D Display (red/green or polarized) requiring goggles or a specialized screen. Alternatively, a physical target, such as an LED mounted in front of the test screen could be used as a convergence target. In contrast to measurement of coordination on a flat screen, such 3D technologies would have to be custom built, installed and calibrated. Empirical assessment of the data from such convergence/divergence measures would have to be tested to see if there was substantial diagnostic value in the additional equipment required.

**Population Norms, Confounds and Clinical Conditions to be tested**

In the initial studies of the normal populations, subjects with normal, fully developed oculomotor systems should be tested:

- Adults 18-40 (before presbyopia)

- Normal Active Convergence/Accommodation Angles or history of strabismus

- Emmetropia with no need for corrective lenses.

- No history of eye disease or eye surgery.

- No confounding medical conditions such as stimulant or sedating medications, sleep disorders or other neurologic conditions, including headache, vertigo, stroke, TIA, Motor control disorders (such as Parkinson’s disease), cerebellar disorders, schizophrenia, demyelinating disease, cognitive disorders (such as Attention Deficit Hyperactivity Disorder or dementia).

 Studies of the norms of binocular coordination can be carried out on existing systems that are FDA approved for conventional assessment of oculomotor control. These include systems that can sample at 100 Hz or higher for high temporal resolution of saccadic movements and that can give good measures of saccade velocity. An important consideration will be sampling intervals of both eyes and how well they are coordinated. Modern oculomotor systems include technologies that use remotely mounted cameras to examine facial features and detect eye movements with no equipment mounted on the test subject. Such systems may have lower sampling rates and lower spatial resolution of eye movement activity. By examining the natural variability of binocular coordination with high resolution head mounted systems, the resolution required for acceptable performance by remote camera systems will be determined. Ultimately technologies that can measure eye movements in freely moving subjects with little or no body worn gear will be achieved.

**Medical conditions with Binocular Variability**

As population norms of binocular variability are being established, examination of subjects with a variety of medical conditions will help establish variations that occur pathologically, in acute disease, during treatment and in recovery. Medical conditions to be examined include oculomotor disorders, visual-vestibular disorders and central nervous system disorders. 

**Oculomotor Disorders**

People with chronic and acute strabismus are clear candidates for examination of binocular coordination. The static deviations of alignment can be compared with the binocular coordination features. As we determined in studies of recovery from alignment changes, there are rapid recovery systems for binocular re-alignment, but these systems have limits to the deficits that can be measured (7,8). New studies of the study of binocular alignment modifications can be used to assess strabismus treatment regimes, but also for more subtle difficulties, such as difficulty with “focus” on prolonged viewing of display images or written text. Indeed, binocular coordination metrics should be used for evaluation of orthoptic exercises and even for their prescription and treatment plans (4).

**Visual-Vestibular Disorders**

**Figure 1:**
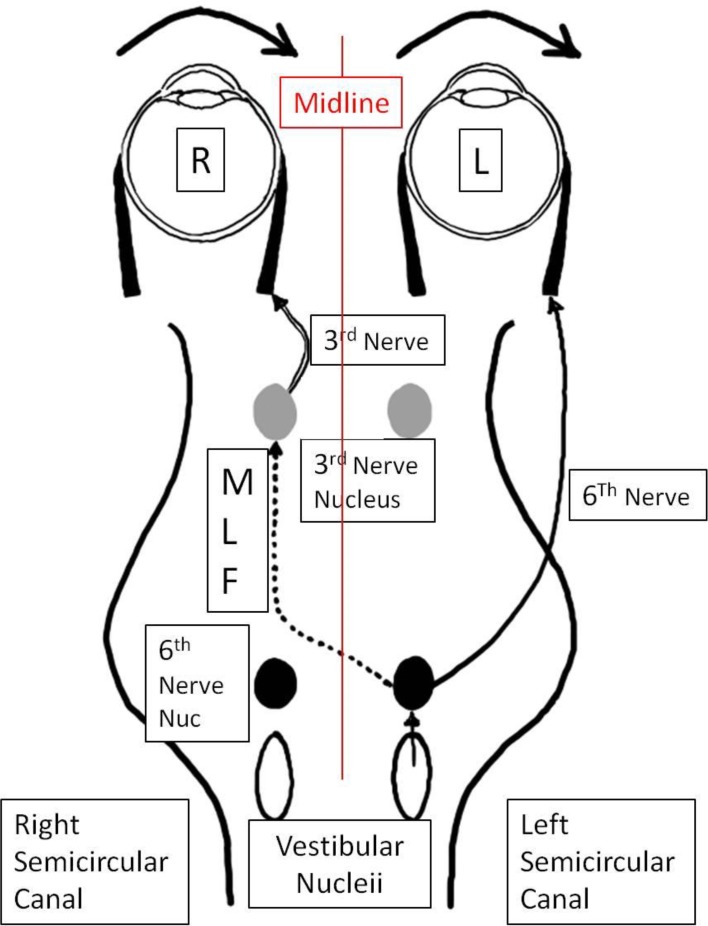
Brainstem structures for VOR eye movement control. The system is Mirror Symmetric across the midline, thus the “push-pull” structures for left and right eye movements of each eye are not the same.

As has been demonstrated (9) the body has two VORs, one for each eye. Most assessment of visual-vestibular function looks at the VOR or nystagmus of just one eye, tacitly making the assumption that the other eye is “along for the ride”. However, because of the mirror symmetry of the body and the visual-vestibular pathways, by definition, when there is a “push-pull” set of signals driving eye movements, such as during the VOR, there are different circuits driving each eye (Figure 1). 

 Thus disorders of the vestibular system, such as Meniere’s Syndrome, Vestibular Neuritis or Migraine related vertigo all have the potential to differentially affect the eye movement driving signals of the two eyes as the vestibular apparatus of one ear is damaged through disease. Thus binocular coordinate metric deviations from normal population values can be established during the acute and recovery phases of these diseases. Interestingly, there is a vestibular disorder that can be treated within minutes: Benign Paroxysmal Positional Vertigo (BPPV) (10). There are acute changes in the VOR and induction of nystagmus provoked by a position change. In addition many patients describe an instant relief of imbalance sensations when the treatment is applied. Given the strong dysfunction of the vestibular apparatus during BPPV and the near-immediate relief of the condition with the canalith-repositioning maneuver, measurements of binocular coordination, before, during and after treatment of BPPV will give important information regarding the variability of coordination.

**Central Nervous System Disorders**

It can well be expected that central motor control disorders (Parkinson’s Disease) and even central visual disorders (Migraine Aurae) should have measureable effects on binocular coordination especially during acute phases of these diseases. Such conditions should be studied for measurements of deviation from population norms. However, what may be of great interest is measurement of conditions that are perhaps more subtle in presentation and perplexing in terms of central effects that have complaints such as “eye-strain” or “difficulty focusing”. Such conditions include chronic headaches, post-concussion syndromes and even Attention Deficit Hyperactivity Disorder (ADHD) and autism. Determination of the binocular coordination metrics during these conditions may ultimately demonstrate subtle, yet large deficits that would both could be diagnostic as well as provide explanations of visual system complaints. Further, metrics of binocular coordination could plausibly be useful in the evaluation of positive effects of therapy and even the onset of adverse effects of medications.

**Models of Binocular Eye Movement Control**

Highly quantitative, high quality models of the control of movements of both eyes have been established from animal models and matching of human eye movement behaviors to the animal work. The control systems of basic reflexes of the VOR and the high complex target selection systems such as saccadic refixations and pursuit targets have been localized and the relevant neuronal signals have been measured with high precision. As mentioned above, the brain is mirror symmetric. Integrated models of control for the various eye movement control systems have been built (11). Importantly, these models are not just wire-frame connections between centers, but quantitative signal models of 3D control. Using these models it should be possible to predict effects of a variety of deficits, including peripheral sensory damage, changes in eye muscle strength through disease and medical treatment and even more diffuse CNS effects such as drug intoxication on binocular coordination. 

Importantly, the coordination of the eyes can be assessed not only in the pure horizontal plane, but also in the vertical and even cyclotorsional dimensions. Again the models of oculomotor control and the data from research should give us some indication of the variation in binocular coordination. 

**Technical Issues**

The initial characterization work of determining population norms of binocular coordination should be done in reasonably constrained conditions. As described above, the standard oculomotor testing regimes should be used with carefully screened test subjects. However, more natural testing conditions should also be included in a testing program. Systems that are un-obtrusive and potentially not mounted on the subject should be considered to enable determination of metrics during natural work conditions, such as computer screen work or even light manual activities on a bench top. It is conceivable that oculomotor metrics in these realms could be important predictors of ability to work and concentrate. 

## CONCLUSION

Binocular Coordination variability is an interesting statistic that can be sensitively assessed. It can be assessed in the velocity domain without extensive gaze position recalibration, thus enabling recording over long intervals. Variability can thus be used as a robust, long term eye movement parameter with minimal intrusiveness to the subject. Establishment of population norms in carefully selected subjects should result in closely constrained ranges of normal function. Then examination of a variety of eye, vestibular and CNS conditions can be measured to determine their characteristic patterns of deviations from the norm. Criticism of some neurologic tests involving reading or other visual-motor activities has been made because the eye movement norms are not know and the deviation from the norms not established in medical conditions such as post-concussion syndrome (12). With the advent of handheld devices that can crudely measure eye activity (http://www.samsung.com/us/guide-to-galaxy-smart-devices/galaxy-s-4-smartphone.html?cid=ppc-) and more advanced systems that are head worn (http://en.wikipedia.org/wiki/Google_Glass), eye movement behavior will become a routine feature of human performance and disease assessment. Measurement and comparison of the activity of both eyes should be required for these assessments.

## References

[1] Tsai YF, Viirre E, Strychacz C, Chase B, Jung TP (2007). Task performance and eye activity: predicting behavior relating to cognitive workload. Aviat Space Environ Med.

[2] Viirre E, Van Orden K, Wing S, Chase B, Pribe C, Taliwal V, Kwak J (2004). Eye movements during visual and auditory task performance Society of information display. Digest of Technical Papers.

[3] Bucci MP, Brémond-Gignac D, Kapoula Z (2008). Poor binocular coordination of saccades in dyslexic children. Graefes Arch ClinExpOphthalmol.

[4] Gaertner C, Bucci MP, Ajrezo L, Wiener-Vacher S (2013). Binocular coordination of saccades during reading in children with clinically assessed poor vergence capabilities. Vision Res.

[5] Bahill AT, Clark MR, Stark L (1975). The main sequence, a tool for studying human eye movements. Mathematical Biosciences.

[6] Rothenberg SJ, Selkoe D (1981). Specific oculomotor deficit after diazepam. I. Saccadic eye movements. Psychopharmacology (Berl).

[7] Viirre E, Cadera W, Vilis T (1987). The pattern of changes produced in the saccadic system and vestibuloocular reflex by visually patching one eye. J Neurophysiol.

[8] Viirre E, Cadera W, Vilis T (1988). Monocular adaptation of the saccadic system and vestibulo-ocular reflex. Invest Ophthalmol Vis Sci.

[9] Viirre E, Tweed D, Milner K, Vilis T (1986). A reexamination of the gain of the vestibuloocular reflex. J Neurophysiol.

[10] Epley JM (1992). The canalith repositioning procedure: for treatment of benign paroxysmal positional vertigo. Otolaryngol Head Neck Surg.

[11] Ranjbaran M, Galiana HL (2012). The horizontal angular vestibulo-ocular reflex: a non-linear mechanism for context-dependent responses. ConfProc IEEE Eng Med Biol Soc.

[12] Galetta MS, Galetta KM, McCrossin J, Wilson JA, Moster S, Galetta SL, Balcer LJ, Dorshimer GW, Master CL (2013). Saccades and memory: baseline associations of the King-Devick and SCAT2 SAC tests in professional ice hockey players. J Neurol Sci.

